# Expression of Urocortin 3 mRNA in the Central Nervous System of the Sea Lamprey *Petromyzon marinus*

**DOI:** 10.3390/biology10100978

**Published:** 2021-09-28

**Authors:** Daniel Sobrido-Cameán, Ramón Anadón, Antón Barreiro-Iglesias

**Affiliations:** 1 Department of Functional Biology, CIBUS, Faculty of Biology, Universidade de Santiago de Compostela, 15782 Santiago de Compostela, Spain; ds918@cam.ac.uk (D.S.-C.); ramon.anadon@usc.es (R.A.); 2 Department of Zoology, University of Cambridge, Cambridge CB2 3EJ, UK

**Keywords:** urocortin 3, in situ hybridization, brain evolution, striatum, agnathans

## Abstract

**Simple Summary:**

Neuropeptides are small proteins produced and released by neurons. Neuropeptides are key mediators in the communication between neurons. Urocortin 3 is an important neuropeptide that is involved in stress-related behaviors. In this study, our aim was to determine in which regions of the lamprey brain urocortin 3 is expressed. The study of the distribution of neuroregulatory peptides in lamprey species helps in understanding their early evolution of neuropeptidergic systems in vertebrates because lampreys are one of the most ancient vertebrate groups. The sea lamprey life cycle lasts between five and nine years, and it includes a long larval stage and a transformation to the adult stage. We analyzed the organization of urocortin 3-expressing neuronal populations in the brain of larval and adult sea lampreys to establish whether this neuropeptide is expressed differentially in these two widely different phases of the sea lamprey life cycle. Our results revealed important differences between larval and adult stages. Moreover, comparing our results with the distribution of urocortin 3 reported in other vertebrate groups also revealed a poor evolutionary conservation of urocortin 3 expression in vertebrates. Our work sheds light on the evolution and role of urocortin 3 in vertebrates.

**Abstract:**

In this study, we analyzed the organization of urocortin 3 (Ucn3)-expressing neuronal populations in the brain of the adult sea lamprey by means of in situ hybridization. We also studied the brain of larval sea lampreys to establish whether this prosocial neuropeptide is expressed differentially in two widely different phases of the sea lamprey life cycle. In adult sea lampreys, Ucn3 transcript expression was observed in neurons of the striatum, prethalamus, nucleus of the medial longitudinal fascicle, torus semicircularis, isthmic reticular formation, interpeduncular nucleus, posterior rhombencephalic reticular formation and nucleus of the solitary tract. Interestingly, in larval sea lampreys, only three regions showed Ucn3 expression, namely the prethalamus, the nucleus of the medial longitudinal fascicle and the posterior rhombencephalic reticular formation. A comparison with distributions of Ucn3 in other vertebrates revealed poor conservation of Ucn3 expression during vertebrate evolution. The large qualitative differences in Ucn3 expression observed between larval and adult phases suggest that the maturation of neuroregulatory circuits in the striatum, torus semicircularis and hindbrain chemosensory systems is closely related to profound life-style changes occurring after the transformation from larval to adult life.

## 1. Introduction

A 41-amino-acid corticotropin-releasing factor or hormone (CRF; CRH; CRH1) purified from the hypothalamus was characterized by its ability to stimulate the release of corticotropin from pituitary cells [1]. In addition to its role as a releasing hormone, this peptide also acts as a neuromodulator in various brain regions, and accordingly the names CRF or CRH do not refer to the full range of its functions as a neuropeptide [2]. More recently, a family of CRH1-related peptides (i.e., CRH2, urotensin or urocortins) were identified in vertebrate and invertebrate species [3,4,5,6,7,8,9]. In invertebrate chordates such as amphioxus and ascidians, only one CRH-family gene was reported [10]. In vertebrates, however, the two rounds of whole-genome duplication led to multiple CRH/urocortin (Ucn) genes. It was proposed that a vertebrate ancestor originated two CRH-like genes that then led to two distinct gene lineages, one group coding for CRH1, CRH2 and Ucn/urotensin (Ucn1), and the other coding for urocortin 2 (Ucn2) and urocortin 3 (Ucn3 or stresscopin) [10,11]. Peptides of these two groups interact with two types of CRH receptors (CRHRs), those of the first group with type 1 (CRHR1), and those of the second group with type 2 (CRHR2) receptors [12]. An additional independent duplication of the CRH1 gene (CRHa and CRHb) was reported in some teleosts [13].

The distribution of CRH-type peptides and receptors was studied in the brain of mammals and some other vertebrates with immunohistochemistry, in situ hybridization and biochemical methods. In the rat brain, the distribution of these peptides was observed in distinct neuronal populations for each of the peptides: distributed in hypothalamic populations (CRH; [13]), in the Edinger–Westphal nucleus (Ucn1; [14]) or in several brain regions (Ucn3; [15,16]). The expression of some CRH-family genes was also reported in the brain of some ray-finned fish (zebrafish [17]; medaka [18]; spotted gar [19]; cichlid fish [11,13]; Siberian sturgeon [20]), frogs [21,22] and birds [23]. However, little is known about the brain distribution of peptides of the CRH family in agnathans, excepting the distribution of CRH-immunoreactivity in a hagfish [24]. In lampreys (an agnathan), the CRH sequence was identified from a genome analysis, and it was shown to elevate the levels of a cortisol precursor (S), which is the putative corticosteroid mediating stress responses [25]. The sea lamprey sequences of urotensin I and Ucn3 were identified more recently [9]. Recently, the five CRH-family genes were identified (Crh1, Crh2, Ucn1, Ucn2 and Ucn3) in genomes of two lamprey species (*Petromyzon marinus* and *Lethenteron camtschaticum*; [26]). However, the expression of these genes in specific regions of the lamprey brain is not known.

The study of the distribution of neuroregulatory peptides in lamprey species may help in understanding their early evolution in vertebrates because lampreys (jawless vertebrates) are the extant outgroup of the other (jawed) vertebrates. Ucn3 is particularly interesting because in the mouse, it is expressed in brain regions involved in stress-related behaviors (paraventricular nucleus, rostral perifornical area, amygdala, bed nucleus of the stria terminalis) [27,28,29,30]). Here, we studied the distribution of Ucn3 in the brain of the sea lamprey (*Petromyzon marinus*) in two very different life phases, larval (a burrowing, filter-feeding, blind phase lasting several years) and adult (parasitic life-style, feeding actively on fish and with developed vision), that show important differences in some brain centers [31].

## 2. Materials and Methods

### 2.1. Animals

Larvae (around 100 mm in body length, *n* = 11), downstream young adults (post-metamorphic juveniles; *n* = 3) and upstream (sexually mature; *n* = 3) migrating adults of the sea lamprey, *Petromyzon marinus* L., were used in this study. Larvae and downstream migrating young adults were collected from the River Ulla (Galicia, Spain) with permission from the Xunta de Galicia. Upstream migrating adults were acquired from local suppliers. Before all experiments, animals were deeply anesthetized with 0.1% tricaine methanesulfonate (MS-222; Sigma-Aldrich, St. Louis, MO, USA) in fresh water and killed by decapitation. All experiments were approved by the Bioethics Committee of the University of Santiago de Compostela and the Xunta de Galicia and were performed in accordance with European Union and Spanish guidelines on animal care and experimentation.

### 2.2. Cloning and Sequencing of the Sea Lamprey Ucn3 cDNA

cDNA was obtained from the brain of 8 larvae as previously described (Sobrido-Cameán et al., 2019). For polymerase chain reaction (PCR) cloning, specific oligonucleotide primers (forward: 5′-CCTGGACGTTCCCACCAACA-3′; reverse: 5′-GGACCTGGAACCTGGAACCC-3′) were designed based on the P. marinus Ucn3 transcript sequence deposited in GenBank with accession number KX446867.1 [9]. The amplified fragments were cloned into pGEM-T easy vectors (Promega, Madison, WI, USA) and sequenced by GATC Biotech (Cologne, Germany) using Sanger sequencing. This confirmed that we cloned a 321 bp cDNA sequence of the previously identified sea lamprey Ucn3.

### 2.3. In Situ Hybridization

Templates for in vitro transcription were prepared by PCR amplification of the cloned Ucn3 cDNA fragment using the primers mentioned above. In this case, the reverse primers included the sequence of the universal T7 promoter (TAAGCTTTAATACGACTCACTATAGGGAGA). For the generation of sense probes, the sequence of the T7 promoter was included in the forward primers. Digoxigenin (DIG)-labeled riboprobes were synthesized using the amplified fragments as templates and following standard protocols using a T7 polymerase (Nzytech, Lisbon, Portugal). In situ hybridization (ISH) experiments with the sea lamprey Ucn3 DIG-labeled riboprobe (1 µg/mL) were performed as previously described for other neuropeptide riboprobes in the sea lamprey [32,33,34] using cryostat transverse sections of larval, juvenile and adult brains/rostral spinal cords. No staining was observed in the sections incubated with the sense probe (not shown).

### 2.4. Image Acquisition and Montage

Photomicrographs of representative sections were taken with an Olympus photomicroscope (AX-70; Provis) equipped with a digital camera (Olympus DP70, Tokyo, Japan). Representative schematics, drawings and plates of photomicrographs were generated with CorelDRAW 12 (Corel, Ottawa, ON, Canada).

## 3. Results

### 3.1. Adult Lampreys

**Figure 1:**
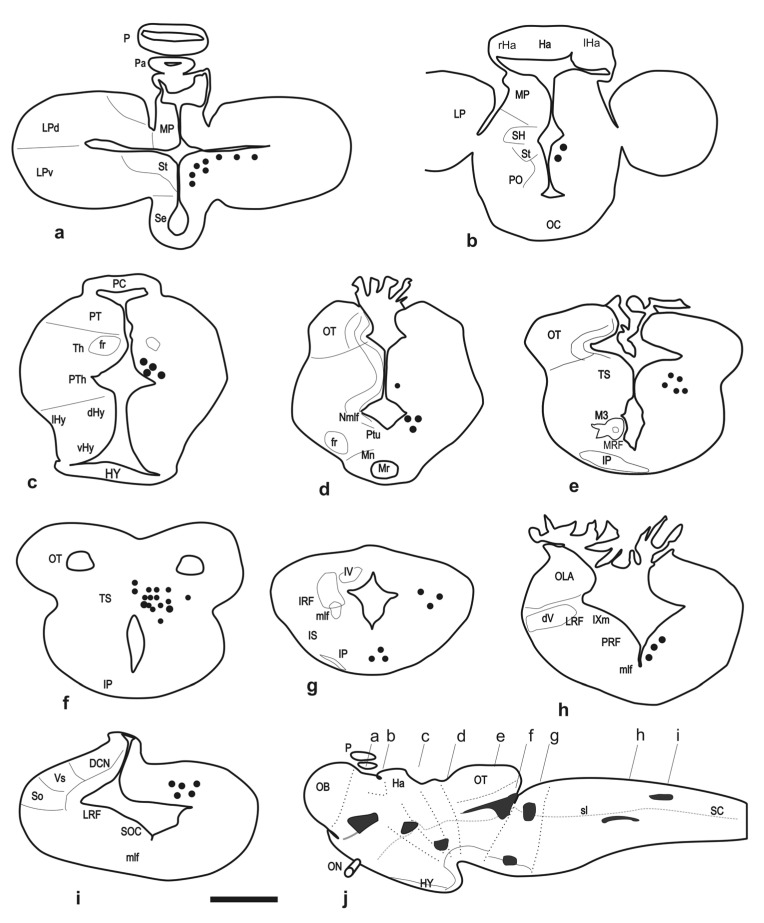
Schematic drawings of transverse sections (**a**–**i**) of the brain of a juvenile sea lamprey showing the distribution of Ucn3 positive neurons (black dots) on the right side of sections. The anatomical structures are indicated on the left side. (**j**), Lateral view of the lamprey brain showing the location of Ucn3 positive populations (black areas) and the level of the transverse sections shown in (**a**–**i**). For abbreviations, see the list. Scale bar, 250 µm (applies from **a**–**i**).

The expression of the Ucn3 mRNA in the sea lamprey brain was studied by in situ hybridization in upstream migrating adults, young post-metamorphic adults (juveniles) and larvae. In adults, expression was found in more regions than in larvae, extending from the telencephalon to the medulla (Figure 1). These neuronal populations will be described following a rostro-caudal direction. Since no differences were observed between juveniles and mature adults, expression data from both stages will be described together in this section.

**Figure 2:**
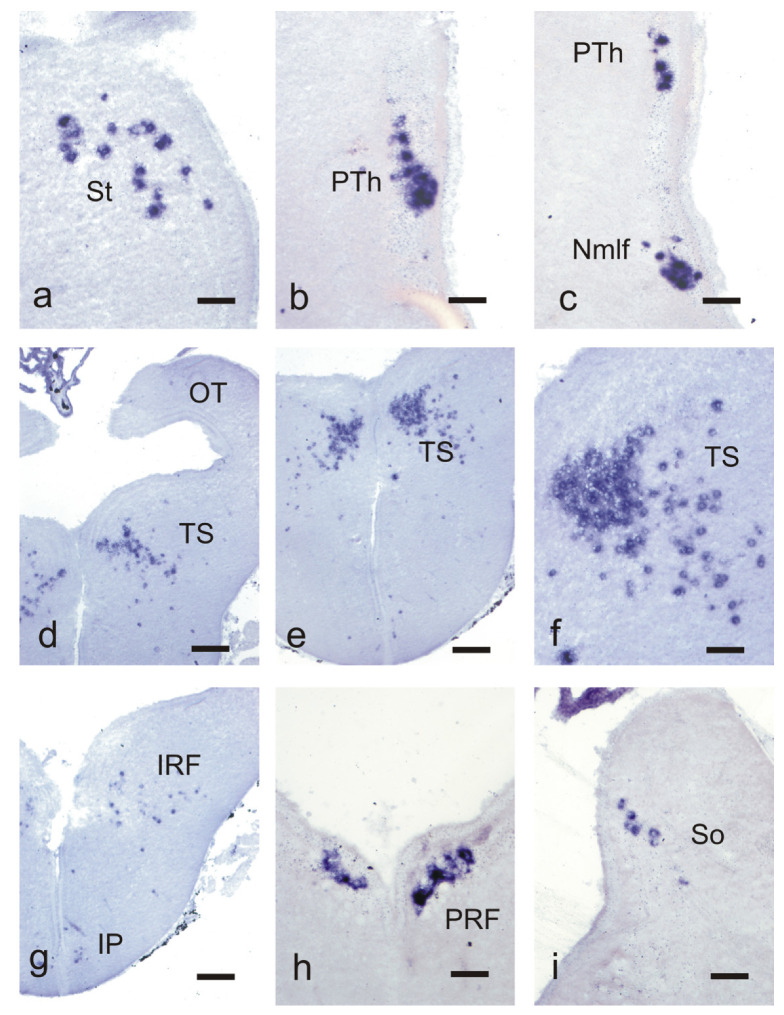
Photomicrographs of transverse sections of the brain of juvenile/adult sea lampreys showing Ucn3 positive neurons in the striatum (**a**), prethalamus (**b**), nucleus of the medial longitudinal fascicle (**c**), torus semicircularis (**d**–**f**), isthmus (**g**), posterior rhombencephalic reticular formation (**h**) and nucleus of the solitary tract (**i**). For abbreviations, see the list. Scale bars, 100 µm (**a**–**c**,**f**,**h**,**i**), 250 µm (**d**,**e**,**g**).

In the telencephalon, a prominent population expressing Ucn3 was observed in the striatum forming an arch parallel to the ventricle (Figure 1a,b,j and Figure 2a). In the central regions of the striatum, up to 30 Ucn3 positive cells per section were observed. No positive cells were observed in the preoptic region or the hypothalamus. In the diencephalon (comprising the derivatives of prosomeres 1 to 3), two populations of Ucn3 positive cells were observed (Figure 1c,d,j). The most numerous formed a column in the prethalamus (formerly the ventral thalamus; alar region of prosomere [3,35]) near the ventral tier of the thalamus (Figure 1c and Figure 2b). A second population of sparse Ucn3 positive cells was observed in the nucleus of the medial longitudinal fascicle (Figure 1d and Figure 2c).

In the midbrain, a large population of Ucn3 positive cells was observed in the caudal torus semicircularis as a bilateral population that joins caudally in the dorsal midline (horseshoe-shaped) (Figure 1e,f,j and Figure 2d–f). Caudally, this cell population ends rostrally to the midbrain-hindbrain boundary. In rostral regions of the torus, Ucn3 positive cells are scarce. This toral population is the largest Ucn3 population in the adult brain.

In the rhombencephalon, four populations of Ucn3 positive cells were observed. The most rostral population was comprised of scattered Ucn3 positive cells located in the isthmic region named as dorsal isthmic gray or isthmic reticular formation, near the tract of the anterior octavomotor nucleus (Figure 1g,j and Figure 2g). A sparse population of Ucn3 positive cells was also found on both sides of the ventral midline in the interpeduncular region (Figure 1g,j and Figure 2g). A third rhombencephalic Ucn3 positive population was located caudally to the octaval nerve entrance in the medial region of the reticular region (posterior rhombencephalic reticular nucleus) over the thick axons of the medial longitudinal fascicle (Figure 1h,j and Figure 2h). The highest caudal Ucn3 positive population was observed in the periventricular location of the alar region at the level of the vagal motor nucleus and was tentatively identified as part of the nucleus of the solitary tract (Figure 1i,j and Figure 2i).

### 3.2. Larval Brain

**Figure 3:**
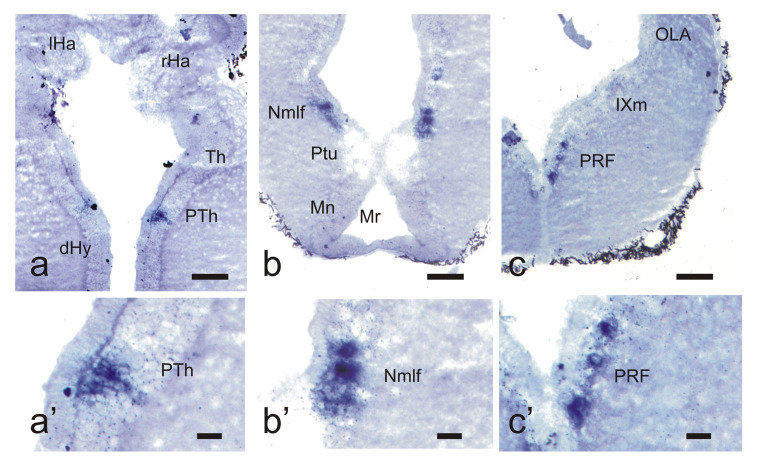
Photomicrographs of transverse sections of the brain of larval sea lampreys showing Ucn3 positive neurons in the prethalamus (**a**,**a’**), nucleus of the medial longitudinal fascicle (**b**,**b’**) and posterior rhombencephalic reticular formation (**c**,**c’**). For abbreviations, see the list. Scale bars, 100 µm (**a**–**c**), 20 µm (**a’**–**c’**).

In the larval brain, the Ucn3 positive populations are restricted to the diencephalon and the rhombencephalon. Unlike in adults, no positive populations were observed in the telencephalon. In the diencephalon, Ucn3 positive neurons were observed in the prethalamus (1 to 3 positive cells per hemisection) (Figure 3a,a’) and more caudally in the nucleus of the medial longitudinal fascicle (1 to 4 cells per hemisection) (Figure 3b,b’). The total number of cells was not assessed, but the cells are much less numerous than in the same nuclei of adults. No positive cells were observed in the midbrain region containing positive cells in adults (torus semicircularis).

In the larval rhombencephalon, Ucn3 positive neurons were only observed in the medial region of the rhombencephalic reticular nucleus caudally to the octaval nerve entrance (posterior reticular formation). There were a few cells located close to the midline near the medial longitudinal fasciculus (Figure 3c,c’) or occasionally in a more lateral position. No Ucn3 positive cells were observed in the isthmus or in the region of the nucleus of the solitary tract.

## 4. Discussion

Here, we report the first study on the distribution of Ucn3 mRNA in the brain of an agnathan, the sea lamprey. Ucn3 expression was analyzed at two very different life stages, adult (juveniles and sexually mature) and larval. A comparison between adults and larvae revealed important developmental changes in Ucn3 expression, with new positive populations appearing in the striatum, torus semicircularis, isthmus and nucleus of the solitary tract after the metamorphosis. Transformation in the sea lamprey is complex, and in the nervous system, it largely affects the visual system including the eye and optic tectum [36,37,38,39,40,41], but also other neural systems such as the medial pallium, cutaneous sensory systems and the olfactory rosette [42,43,44]. Other body organs also suffer important anatomical and physiological changes during transformation to adapt adult lampreys to a new adult life-style (free and ectoparasitic life), which is very different from the blind, burrowing, water-filtering larvae [31]. We are tempted to speculate that metamorphic changes in the Ucn3 system of the sea lamprey brain might be related to lives as adults in a more complex world involving downstream migrations (young lampreys), an active search of preys in the sea, and upstream (sexually mature adults) migrations in the river, as well as complex behaviors related to reproduction in the construction of nests. In this regard, the emergence of Ucn3 positive populations in the adult striatum and torus semicircularis can be related with some higher brain functions. While in situ hybridization revealed, for the first time, specific patterns of Ucn3 gene expression in the sea lamprey brain, this method says little about the connections of these populations and hence on the participation of Ucn3 positive neurons in brain circuits, which can only be hypothesized by a comparison with results of tracing studies in lampreys. Moreover, a comparison with the distribution of Ucn3 in brains of other vertebrate groups, in which some functions of Ucn3 are known, might shed some light on its possible roles in lampreys and will be discussed in the following paragraphs.

In the lamprey telencephalon, a conspicuous group of Ucn3-expressing cells was found in the striatum. The sea lamprey striatum mostly consists of GABAergic neurons [45,46,47], as well as some neurons expressing other neurotransmitters and neuropeptides, e.g., substance P, enkephalin, dopamine; [48,49,50]. The cells expressing Ucn3 are located in the characteristic striatal cell band and could thus co-express GABA or other neurotransmitters, with Ucn3 contributing to neuromodulation by striatal efferent neurons. The main efferent neurons of the lamprey striatum are projections to the ventral part of the lateral pallium [45,46]. Striatal neurons also project to the isthmic GABAergic population that are thought to be the substantia nigra pars reticulata homologue [51]. On the other hand, experimental studies previously revealed that the lamprey striatum receives dopaminergic fibers from a nucleus considered homologous of the substantia nigra pars compacta of mammals, which in lampreys is located in the posterior tubercle [45,46,49,50,51,52,53]. The striatum also receives abundant enkephalinergic and serotonergic fibers [46]. Tracer injections in the striatum also revealed striatal afferent neurons in the olfactory bulb, dorsal and medial pallium, preoptic region, dorsal thalamus (thalamus), ventral thalamus (prethalamus), hypothalamus, isthmus region and trigeminal raphe region [45].

Recent studies in lampreys stressed the great similarity of circuits and the neuronal activity of the striatal system with those of mammals [51]. Present findings with Ucn3 expression in the lamprey striatum suggest, however, the existence of important neurochemical differences with the striatum of mammals, because no expression of Ucn3 was reported in mammalian striatal cells. In the rodent forebrain, Ucn3 positive regions included the medial amygdaloid nucleus, the bed nucleus of the stria terminalis (extended amygdala), the median preoptic nucleus and the rostral perifornical area [15,16]. A role of amygdaline Ucn3 as an anti-stressor neuropeptide was previously reported. If Ucn3 expression in lamprey striatal cells is implicated in similar anti-stress functions, this population might be considered as part of the lamprey limbic system. Studies on mice indicate that the Ucn3 expression in the medial amygdala and that of the receptor CRHR2 are also important for complex social dynamics [16,30]. Equivalences between these prosocial centers in mice with lamprey centers have yet to be reported, and the social life of lampreys is poorly known. Interestingly, the lamprey striatum, as the mouse medial amygdala, receives secondary olfactory projections [45,54], which might be important in mature adults for feeding, reproduction and mate choosing [55]. Recent studies of the olfactory system in the sea lamprey revealed two olfactory pathways via the medial and non-medial parts of the olfactory bulb that project toward the posterior tubercle and motor centers, and to the lateral pallium, respectively [56,57], but a direct relation of these medial and non-medial pathways with the striatum has yet to be deciphered. Although experimental results in the lamprey striatum indicate that it is involved in locomotor control, the possible role of striatal Ucn3 in the modulation of stress/social behaviors should be investigated. No Ucn3 populations were observed in the striatum of the chicken [23] or in the developing zebrafish forebrain [58]. However, Ucn3 expression was reported in cells of two nuclei of the ventral telencephalic area (Vd and Vp) of two advanced teleosts (medaka [18]; cichlid fish [11]), which are considered homologous of the mammalian striatum and/or amygdala. Unlike in the sea lamprey, rodents and chickens, numerous Ucn3-expressing cells were observed in the olfactory bulb of a cichlid fish [11], which indicates that highly divergent patterns of the distribution of this peptide occur in vertebrate telencephalons.

The lack of Ucn3 expression in cells in the preoptic region and hypothalamus of the lamprey is notable and suggests that Ucn3 is not related directly with the hypophysis, which is in contrast with the expression of Ucn3 in the preoptic and/or hypothalamic nuclei of teleosts [11,18]. Hypophysiotropic hypothalamic nuclei do not express Ucn3 in rodents [15,16], which is similar to lampreys.

In the diencephalon of both larvae and adults, two populations of Ucn3 positive neurons were observed, namely in the prethalamus and in the nucleus of the medial longitudinal fascicle. These regions contain neurons that project to the medial and posterior rhombencephalic reticular nuclei, intervening as premotor centers [52], and receive projections from the pallium, striatum, preoptic nucleus, nucleus of the postoptic commissure, thalamus and hypothalamus [59], and this suggests that Ucn3 positive diencephalic populations might be involved in premotor control; however, whether these cells actually project to reticular centers needs to be investigated. Unlike the striatal, toral and highest hindbrain Ucn3 positive populations, which are only detected in adults, those observed in the diencephalon and posterior rhombencephalic reticular formation are present in both larvae and adults, which indicates shared functions in both life stages. An immunohistochemical study in amphioxus using an antibody raised against carp urotensin I revealed a population of neurons just caudal to the brain vesicle [60], i.e., in a region considered homologous to the vertebrate diencephalon [61]. However, it is difficult to compare the expression of the multiple CRH/Ucn systems in the brain of vertebrates with those of chordates such as amphioxus that express a single CRH/Urocortin peptide.

The toral population of Ucn3-expressing neurons of the adult sea lamprey is located rostrally to the mid-hindbrain boundary and the trochlear nucleus. Recently, a roughly similar region ventrocaudal to the optic tectum was identified as the putative substantia nigra pars reticulata homologue [49,50], although these authors do not clarify whether it is in the midbrain or in the hindbrain (isthmus). This region appears to receive striatal projections [40,41,42,43,44,45,46,47,48,49,50]. Moreover, the region depicted by these authors as the midbrain locomotor region might also correspond in part with that occupied by Ucn3-expressing neurons. It is unclear whether the anatomical differences are due to between-species differences (those authors employed *Lampetra fluviatilis* in their studies) or not. Cells in the caudoventral region of the tectum express the dopamine receptor D2 and project to the putative substantia nigra pars compacta [62]. Judging from the reported distribution of these D2 positive cells, it appears probable that Ucn3-expressing cells in this region (caudal midbrain) correspond to the same population. However, we feel that this population is toral, not tectal, because the torus semicircularis extends caudally in this position, protruding on the surface of the brain and replacing the tectum at this level [40], in a way similar to that reported in *Xenopus laevis* [63]. A study on adult sea lampreys revealed that this “caudal pole of the optic tectum” receives mainly octavolateral fibers, but no optic fibers [64], indicating that the identity of the caudal region of the lamprey torus was probably misidentified as the optic tectum. The torus lateralis of larval sea lampreys receives afferent axons, among others, from the octavolateralis region as well as from the prethalamus (ventral thalamus) and striatum [65]. A recent study on tilapia revealed that the mechanosensory lateral line system provides relevant information to conserved decision-making centers of the brain [66]. With regards to the possible significance of the toral Ucn3 positive population, it may be hypothesized that electrosensory and/or mechanosensory information coming via the secondary octavolateralis projections on Ucn3 toral cells would be important in social life or predation in adult lampreys. Since these behaviors are not developed in larvae, the absence of Ucn3 expression in the toral structures of larvae might be related with a lack of maturation of toral circuits. Although studies of changes in brain structures during lamprey metamorphosis are mostly centered on the visual system [36,37,67], it appears that the caudal alar midbrain also demonstrates a notable development during the metamorphosis, which deserves further investigation. Whereas the inferior colliculus of mice (the torus semicircularis homologue) does not contain any Ucn3 populations, a study of transgenic mice revealed that it receives specific projections from Ucn3-expressing neurons of the superior paraolivary nucleus [16]. These projections may be related to the modulation of the acoustic startle reflex, which is considered to reflect aspects of anxiety. The expression of Ucn3 was previously reported in the optic tectum of two teleosts, the medaka [18] and a cichlid fish [11], which is unlike the lack of tectal Ucn3 expression observed in lampreys (present results), birds [23] and mammals [15,16], and reveals again divergent evolutionary patterns of expression of this peptide within vertebrates.

In the rat hindbrain, Ucn3 positive cells were detected mainly in the superior paraolivary nucleus [15] and, in the mouse, also in the parabrachial nucleus [16]. Ucn3 expression in the parabrachial nucleus was also reported in chickens [23]. In contrast, in the sea lamprey rhombencephalon, Ucn3 appears more widely expressed than in the rodent hindbrain, with small populations of Ucn3 positive neurons located in the dorsal isthmus, the interpeduncular nucleus region, the posterior rhombencephalic reticular nucleus and the putative nucleus of the solitary tract. The expression of Ucn3 in some posterior rhombencephalic reticular nucleus neurons may suggest a modulatory function for these population. The reticular formation of lampreys is activated by the medial olfactory pathway via the medial olfactory bulb and the posterior tubercle [68]. Since in the larval lamprey hindbrain Ucn3 cells were only observed in the posterior rhombencephalic reticular nucleus, the other Ucn3 positive hindbrain populations observed in adults probably are related with some other aspects of the adult life cycle. The scattered population observed in the dorsal isthmus might correspond to the population reported in the parabrachial region of mice [16], which is related to taste chemosensory pathways. A recent study in the sea lamprey reported the taste bud and solitary chemosensory cells projections via branchiomeric nerves to the solitary tract nucleus and to the isthmic region medial to the anterior octavomotor nucleus [43]. In adult sea lampreys, these receptors are responsive to trout water and some amino acids, and probably intervene in the feeding process of adult sea lampreys. Chemosensory projections might contact Ucn3 positive taste centers in the rhombencephalon of adult sea lampreys, and thus this peptide might modulate some aspects of feeding during adult life. However, possible projections of these cells and hence the higher order nuclei of this taste system are not known. In any case, Ucn3 is not expressed in the taste system in lamprey larvae, in line with that which was indicated above for the olfactory system. In chickens, Ucn3 expression was reported in the glossopharyngeal motor nucleus [23], which was not observed in rodents or sea lamprey. With regards to teleost fishes, a most notable difference with lampreys and rodents is the finding of Ucn3 expression in the granular layer of the cerebellum [11,18]. In chickens, Ucn3 is also expressed in the cerebellum, though it was not detected above background by in situ hybridization [23].

## 5. Conclusions

This in situ hybridization study of the Ucn3-expressing neuronal populations in the brain of the sea lamprey reveals that this neuropeptide is expressed differentially in two widely different phases of the lamprey life cycle. In adult sea lampreys, but not in larvae, Ucn3 is expressed in the striatum, torus semicircularis, isthmic reticular formation, interpeduncular nucleus and nucleus of the solitary tract. These important differences in Ucn3 expression between larval and adult phases suggest that the neurochemical maturation of higher neuroregulatory circuits is closely related to life-style changes occurring after transformation. With regards to larval sea lampreys, the only nuclei showing Ucn3 expression, namely the prethalamus, the nucleus of the medial longitudinal fascicle and the posterior rhombencephalic reticular formation, are shared with adults and might be related to motor control. A comparison with the distribution of Ucn3 reported in other vertebrate groups also revealed a poor evolutionary conservation of Ucn3 expression in vertebrates.

## Data Availability

Data are contained within the article, and materials can be requested from the authors upon reasonable request.

## Abbreviations

DCN	dorsal column nucleus
dHy	dorsal hypothalamus
dV	descending trigeminal tract
fr	fasciculus retroflexus
Ha	habenula
HY	hypophysis
IP	interpeduncular nucleus
IRF	isthmic reticular formation
IS	isthmus
IV	trochlear nucleus
IXm	glossopharyngeal motor nucleus
lHa	left habenula
lHy	lateral hypothalamus
LP	lateral pallium
LPd	lateral pallium, dorsal part
LPv	lateral pallium, ventral part
LRF	lateral reticular formation
M3	mesencephalic M3 Müller cell
mlf	medial longitudinal fascicle
Mn	mamillary nucleus
MP	medial pallium
Mr	mamillary recess
MRF	mesencephalic reticular formation
Nmlf	nucleus of the medial longitudinal fascicle
OB	olfactory bulb
OC	optic chiasma
OLA	octavolateralis area
ON	optic nerve
OT	optic tectum
P	pineal organ
Pa	parapineal organ
PO	preoptic nucleus
PRF	posterior rhombencephalic reticular formation
PT	pretectum
PTh	prethalamus
Ptu	posterior tubercle
rHa	right habenula
SC	spinal cord
Se	septum
SH	subhippocampal lobe
sl	sulcus limitans
So	nucleus of the solitary tract
SOC	spinooccipital nerve nucleus
St	striatum
Th	thalamus
TS	torus semicircularis
vHy	ventral hypothalamus
Vs	descending trigeminal nucleus
